# Functional Ingredients based on Nutritional Phenolics. A Case Study against Inflammation: *Lippia* Genus

**DOI:** 10.3390/nu11071646

**Published:** 2019-07-18

**Authors:** Francisco Javier Leyva-Jiménez, Jesús Lozano-Sánchez, María de la Luz Cádiz-Gurrea, David Arráez-Román, Antonio Segura-Carretero

**Affiliations:** 1 Functional Food Research and Development Center, Health Science Technological Park, Avenida del Conocimiento s/n, E-18100 Granada, Spain; 2 Department of Food Science and Nutrition, University of Granada, Campus of Cartuja, 18071 Granada, Spain; 3 Department of Analytical Chemistry, Faculty of Sciences, University of Granada, Fuentenueva s/n, E-18071 Granada, Spain

**Keywords:** *Lippia*, phenolic compounds, anti-inflammatory, pro-inflammatory mediators, cytokines, prostaglandin, NF-*κ*B

## Abstract

Epidemiological studies have reported convincing evidence that natural dietary compounds may modify inflammation, it being an important event described in the pathophysiology of age-related infirmity. Among different dietary components, nutritional phenolics have demonstrated links to a lower risk of inflammation in the most common degenerative and chronic diseases. In this way, the healthy potential of phenolics against inflammation and the emergence of new functional ingredients have caused an enhancement of nutraceutical and functional food formulation. The present review focuses on: (a) nutritional phenolics and their effects on inflammation and (b) functional ingredients based on phenolic compounds with anti-inflammatory properties. Furthermore, the emerging interest in health-promoting products by consumers has caused an increase in the demand for functional products and nutraceuticals. Additionally, this review includes a case study of the *Lippia* genus, which has shown anti-inflammatory effects claiming to be a natural alternative for the management of this physiological disorder. This report is a practical tool for healthcare providers.

## 1. Introduction

Inflammation has recently been claimed to be a relevant aspect of the pathophysiology of age-related ailments and it has been considered an important factor in causing chronic diseases of industrialized societies, including type 2 diabetes mellitus, cardiovascular disease, Alzheimer’s disease, and many types of cancer [1]. Inflammations and infection disorders bring with an up-regulation of different enzymes and signaling proteins in injured tissues and cells. Inflammatory cells are able to generate and release reactive oxygen species (ROS) and reactive nitrogen species (RNS) such as •O_2_^−^ (superoxide anion), •OH (hydroxyl radical), H_2_O_2_ (hydrogen peroxide), nitric oxide (NO), and ^1^O_2_ (singlet oxygen). The release of ROS and its accumulation in cells may lead the inflammation state. Among the pro-inflammatory enzymes, nitric oxide synthase (iNOS) and cyclooxygenase (COX), are the main ones responsible for increasing the levels of nitrogen monoxide (NO) and prostaglandin E2 (PGE2) which are well-known inflammatory compounds related to several chronic diseases.

Natural dietary intake may become an alternative to preventing or alleviating this disorder, since the intake of natural food contains healthy compounds which seem to play a relevant role in inflammation events. For instance, the intake of micronutrients such as magnesium, polyunsaturated fatty acids (PUFAs), monounsaturated fatty acids (MUFAs) and different groups of phenolics have demonstrated a decrease in the level of pro-inflammatory markers in serum. Nevertheless, these pro-inflammatory markers can be increased by an excessive intake of saturated fatty acids (SFA), trans-fatty acids (TFA) or a high glycemic index carbohydrates [1]. These outcomes have shown the significant influence of dietary patterns on inflammation processes. In this sense, other studies have associated the Mediterranean dietary pattern, which is rich in fresh fruit and vegetables, whole grains and olive oil, with lower levels of pro-inflammatory markers [2].

Among different dietary components, phenolic compounds, including phenolics acids, hydroxycinnamic acids and flavonoids, including proanthocyanidins, have been shown to be related to a lowered risk of most common degenerative and chronic diseases. Their antioxidant and anti-inflammatory activities have been largely attributed to the singular chemical structures [3]. In this sense, medicinal plants have been traditionally used for human health and the evolution of the understanding of molecular aspects of disorders have led to the establishment of new therapeutic targets from plants. As an example, the genus *Lippia* is a perennial plant that belongs to the Verbenaceae family. This genus, which contains verbascoside as a major phenolic component, has been reported to provide several potential pharmacological applications which involve inflammatory processes [4]. Recently, the European Union has listed extracts from plants of the *Lippia* genus, specifically *Lippia citriodora* extracts as new food, enabling its utilization as an ingredient (Commission Implementing Regulation (CIR), European Union (EU) 2017/2470 of 20 December 2017) and opening up a huge range of possibilities to healthcare providers.

For this reason, different extraction and isolation procedures have been developed to retrieve the responsible compounds from natural sources in order to reduce the necessary amounts of food that provide the desired beneficial effect. Moreover, different encapsulation methods have been implemented in order to increase the bioavailability of some phenolics with the aim of developing high value products such as functional foods or nutraceuticals [5,6]. These new products could help to reduce the prevalence of certain diseases or could even be used as a therapeutic alternative for the treatment of various ailments.

In this scenario, the aim of this review is to afford a complete overview of the health benefits associated with phenolic compounds and their anti-inflammatory properties. To achieve this goal, this project was divided into three different sections: (a) biosynthesis and chemical structure of phenolics as potential anti-inflammatory molecules; (b) relationship between inflammation and phenolic compounds, including a report of the main in vitro, in vivo, and clinical trial studies; and (c) development of phenolic functional ingredient against inflammation, a case study of *L. citriodora*.

## 2. Biosynthesis and Chemical Structure of Phenolic Compounds

There are more than 300,000 species of plants documented, which synthesize a large number of compounds with different purposes. Broadly, these compounds can be divided into two major groups. On the one hand, the primary metabolites, such as sugars, amino acids or lipid which are necessary for their development, take part in the photosynthetic and respiratory metabolism of the plant, among other functions. On the other hand, secondary metabolites are a heterogeneous group of compounds that are found in specialized cells and they take action in secondary functions such as reproduction, protecting from predators or ultraviolet radiation, or producing sensory and colorimetric characteristics in different fruits, vegetables, and flowers [7,8]. In addition to these secondary metabolites being the focus of many studies in previous decades, especially phenolic compounds, they have also been shown to possess the ability to improve several physiological functions due to their anti-inflammatory, antimicrobial, antioxidant or cardioprotective properties [3,4,5,6].

Most phenolic compounds are synthesized through different metabolic pathways: pentose phosphate pathway, malonate/acetate, shikimate, and phenylpropanoid pathways [9,10]. More than 10,000 different phenolic structures, whose chemical structure consists of at least one aromatic ring with one or more hydroxyl substituents [9], lead from these pathways.

Moreover, phenolic compounds may build complexes by binding with other substituents. Indeed, phenolic compounds are naturally present as conjugates with sugar moieties or linked to other phenolic groups and they may set out functional derivatives as esters or methyl esters [8,11], although it is also commonly found for them to be associated with organic acids, amines or lipids [9], ranging from simple molecules as cinnamic acids to highly polymerized substances [12]. Because of the huge variety of phenolic structures, they are in essence categorized into several classes according to their basic skeleton. In this sense, structure–activity relationship (SAR) studies have shown that structure and substitution pattern of hydroxyl groups are essential for effective free radical scavenging activity [13].

The simplest phenolic compounds are phenolic acids which in turn are subdivided into hydroxybenzoic acids and hydroxycinnamic acids which have C_6_–C_1_ and C_6_–C_3_ as the particular carbon skeleton, respectively. Phenolic acids usually are bound with sugars or organic acid and they are the units of more complex structures as lignins or hydrolysable tannins [14]. For instance, the union of several gallic acid (a hydroxybenzoic acid) molecules give rise to gallotannins whereas gallic acid and hexahydroxydiphenoyl (HHDP) moieties are both subunits of the ellagitannins. Moreover, *p*-coumaric acid, a hydroxycinnamic acid, is an important precursor to build flavonoids, stilbenoids and xanthones. These compounds can be found wild in nature or, likewise hydroxybenzoic acids, bound with sugars or organic acids. In this way, chlorogenic acid also known as caffeoylquinic acid contains caffeic acid and quinic acid moieties, which are broadly identified in coffee [15]. Moreover, coumarins emerge from hydroxycinnamic acids by cyclization and ring closure between the hydroxyl and carboxyl groups [14]. On the other hand, numerous phenolic acids aldehydes are related to the characteristic aroma of some botanicals and belong to the acetophenones group [16]. In this scenario, Nile et al. have reported the capacity of ferulic acid related compounds to inhibit tumor necrosis factor (TNF)-α and interleukin (IL)-6, where gallic acid showed the highest percentage of inhibition in both parameters. Whereas methyl, propyl, and lauryl gallates reported the lowest anti-inflammatory activity against TNF-α and IL-6 [17]. This could be due to the methylation of the OH group which reduces the scavenging activity [18]. In mushrooms, the reduction of NO production was evaluated in order to study the correlations between phenolic compounds and their anti-inflammatory effects. Taofiq et al. reported that cinnamic acid showed the highest anti-inflammatory activity presenting the lowest EC_50_ values (182 ± 16 μM), followed by *p*-hydroxybenzoic (239 ± 29 μM) and *p*-coumaric (442 ± 33 μM) acids, mainly due to the presence of carboxylic group and no OH groups in the benzene ring [19].

Flavonoids represent the most widespread and known phenolic group. Indeed, only this group contains around 8000 structures which are disseminated in many cells and tissues of plants. They present a basic structure based on three rings building a characteristic C_6_–C_3_–C_6_ skeleton. The wide range of flavonoid is due to many modifications that suffer in their structures from hydroxylation, methoxylation or glycosylation and also the linkage of aromatic ring, giving rise to up to thirteen different subgroups [14]. Some flavonoid structures may bind themselves to form proanthocyanidins that are considered another phenolic group.

Modification of flavonoids including hydroxylation, o-methylation, and glycosylation, can alter their metabolic features and affect mechanisms of inflammation. For example, aglycones are more potent antioxidants than their corresponding glycosides. Specifically, the effective bioactivity of flavonoids mainly depends on the 3′,4′-orthodihydroxy configuration in ring B and 4-carbonyl group in ring C. In addition, the presence of a catechol-like structure in ring C and the C2–C3 double bond configured with a 4-keto arrangement also benefits the antioxidant capacity [20,21]. Quercetin and its derivatives have been reported to demonstrate a notable concentration-dependent inhibitory potential towards synthesis of inflammatory mediators [22]. Lesjak et al., pointed out that the number of free hydroxyl groups of flavonoids is not directly proportional to their anti-inflammatory activity, specifically their COX-2 inhibition potential [22].

Tannins are high molecular weight phenolics. Their high weight is associated to their ability to bind with carbohydrates and proteins. Due to the high variety of structure to get bound, tannins are divided into three categories: hydrolysable tannins, condensed tannins and a newgroup called phlorotannins. The phenolics that belong to the first group, gallotannins and ellagitannins, are high molecular weight molecules with a great antioxidant capacity due to the fact that they can have a variable number of gallic acid moieties [23]. Condensed tannins arise from the union of many of these structures achieving a high degree of polymerization (up to 20 moieties or more). Phlorotannins are polymeric structured, based on the union of many phloroglucinol units and depending on the linkage, they may be divided into four classes: phlorotannins, fuhalols and phlorethols, fucols and fucophlorethols, and eckols and carmalols [14].

Finally, lignins are complex structures based on (C_6_–C_3_)_n_ that form wood from the plants and are considered to be the second major substance in plants. They originate from coniferyl alcohol, *p*-coumaric alcohol or sinapyl alcohol in a complex polymerization process [14].

## 3. Inflammation and Phenolic Compounds

Inflammation is now considered to be an important event at the cellular and subcellular level in diseases. Briefly, this process involves some cells, mainly macrophages, which promote the production of pro-inflammatory mediators such as interleukins (IL) (IL-1β, IL-6, IL-8); tumor necrosis factor (TNF)-α, ROS, NO, and prostaglandins (PGs). Their overproduction may be acute or chronic, depending on two factors: (a) the stimulus and (b) the efficiency of the reaction to remove such stimulus or injured tissues. A long inflammation state may cause chronic degenerative diseases such as arthritis and atherosclerosis and it be associated with other diseases such as asthma, Alzheimer’s or cancer [24].

Phenolic compounds are natural antioxidants of the human diet and have a relationship (direct and indirect) with anti-inflammatory activity helping to alleviate the oxidative stress at the cellular level [25]. Indeed, dietary phenolics have been related to the modification of the inflammatory cascade associated with diseases becoming potential targets in the prevention of such conditions [26,27]. In this sense, some of them act as antioxidants helping to inhibit all the mediators with causes of oxidative stress such as quercetin or luteolin, other compounds such as kaempferol or hesperidin, acts obstructing COX-2 decreasing the NO production and consequently alleviating the inflammatory response [28]. Table 1 summarizes the anti-inflammatory action of some dietary compounds.

**Figure 1:**
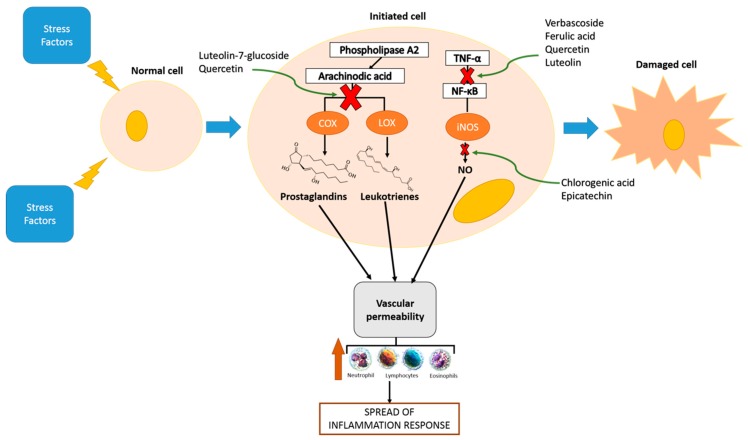
Anti-inflammatory activities of phenolic compounds.

The latest scientific evidence about in vitro and in vivo effects of the phenolic compounds on their anti-inflammatory mechanisms have been developed using isolated compounds or crude phenolic extracts (Figure 1).

Regarding isolated compounds, vanillic acid, a phenolic acid found in plants and fruits, is reported to inhibit carrageenan-induced pro-inflammatory cytokine production (IL-1β, TNF-α, and IL-33) and suppresses activation of nuclear factor kappa-light-chain-enhancer of activated B cells (NF-κB) in mice [45]. In addition, oral administration of myricitrin alleviated toxic liver damage by several mechanisms, including the antioxidant defense system, suppressors of inflammation and inhibitors of profibrotic response. Myricitrin significantly reduced cyclooxygenase-2 (COX-2) and TNF-α overexpression in the liver of tetrachloride (CCl_4_)-intoxicated mice [46]. A study focused specifically on the evaluation of the effect of hydroxytyrosol-supplemented refined olive oil (ROO) at different concentrations (0.5 and 5 mg/kg) was carried out in a rodent model with rheumatoid arthritis. The results showed a down-regulation of COX-2 and iNOS expression in the knee joints of collagen-induced arthritic rats submitted to treatment either with ROO or with hydroxytyrosol-supplemented ROO when compared to positive control. In both tested doses, the serum levels of this proinflammatory cytokine decreased when the rodents were treated with ROO or with hydroxytyrosol-supplemented ROO [47].

However, most research studies conducted on the anti-inflammatory potential of phenolic compounds are focused on crude extracts as well as isolated phenolic compounds. In vitro studies on different extracts such as *Eryngium bourgatii*, *Theobroma cacao* or grape seeds which are rich in phenolics have demonstrated anti-inflammatory effects by reducing the TNF-α-induced intracellular ROS generation and the release of monocyte chemoattractant protein production (MCP-1) [48,49]. In this scenario, since common beans contain phenolic compounds as flavonoids, anthocyanins, flavonol glycosides, tannins, and phenolic acids, a pre-feeding diet with white and black kidney beans causes a reduction of colon mRNA expression of IL-1β and IL-6 and an increase of mRNA expression of IL-10 versus basal diet (BD) + dextran sodium sulfate (DSS)-induced colitis in mice. Moreover, gene expression levels of IL-1β, IL-6, and IL-10 correlated with colon tissue protein levels, where inflammatory cytokine tissue levels were reduced and IL-10 colonic levels were increased by both bean diets compared to BD + DSS. In addition to a reduction in the local inflammatory cytokine profile, pre-feeding kidney bean diets prior to colitis induction also reduced circulating inflammatory cytokine levels [50].

Chandran et al., explored the influence of *Syzygium calophyllifolium* bark methanol extract, enriched in phenolics, on inflammation as a cancer associated symptom [51]. In this study, a cotton pellet implanted inflammation model was used to assess the action of the extract in a chronic inflammatory condition. The results showed that the treated group clearly achieved a decrease in the development of granulation tissue. Furthermore, the tested dose at 200 mg/kg was found effective against granuloma formation revealing an inhibition up to 70% compared to the standard drug indomethacin (57%). These facts demonstrated that *S. calophyllifolium* bark may be a natural alternative to repress the migration inflammatory cells, and to prevent irregular permeability of the blood capillaries. In this way, the chokeberry-concentrate phytochemical profile revealed high content of phenolics including cyanidin-based anthocyanins, proanthocyanidins, and flavonoids, among others. The outcomes pointed out that chokeberry concentrate was able to suppress both the release of TNFα, IL-6, and IL-8 in human peripheral monocytes and to cause the activation of the NF-κB pathway in RAW 264.7 macrophage cells. Additionally, chokeberry showed a synergetic effect with sodium selenite inhibiting activation of NF-κB, cytokine release, and PGE2 synthesis [52].

Moreover, a great number of clinical trials have been conducted to evaluate the potential of dietary phenols on anti-inflammatory functions. Indeed, several phenolic supplementations have been conducted in subjects who have some disorders related to metabolic syndrome, including obesity [53]. Shi et al., reviewed several studies that reported the potential benefits of blueberry extracts on inflammatory mediators [54]. Regarding high dietary polyphenol intake from a Mediterranean diet, a sub study of 1139 high-risk participants was carried out within the PREDIMED trial. After the intervention with a low-fat diet according to the American Heart Association guidelines, the Med diet + extra-virgin olive oil and the Med diet + nuts, participants showed significant changes from base line values in vascular cell adhesion molecule 1 (VCAM-1), intracellular adhesion molecule 1 (ICAM-1), IL-6, TNF-α and MCP-1 concentrations in the three intervention groups. In the covariate analysis with VCAM-1, ICAM-1, IL-6, TNF-α and MCP-1 concentrations at one year as dependent variables, circulating VCAM-1, ICAM-1, IL-6, TNF-α and MCP-1 concentrations decreased in both Med diets and increased in the control diet group [55]. This effect of phenolics has also been investigated in arthritis models; Oliviero et al., demonstrated the ability for plant-derived natural compounds to modulate the pro-inflammatory state which characterize the most prevalent arthropathies [56].

Therefore, it is clear that the modulation of inflammatory pathways, mediators, and genes by ingestion of dietary phenolics plays a relevant role in the prevention, mitigation, and treatment of many chronic diseases. Nevertheless, it is necessary to know the metabolites produced and the influence of environmental factors on metabolism in order to determine the impact of phenolics and their metabolites on health [57]. In this sense, multidisciplinary research has been addressed to develop phenolic ingredients against inflammation which could be a practical tool for healthcare providers. Indeed, there are many commercial nutraceuticals attained from different natural sources which have been tested in induced inflammation models with promising results and some of them are listed in Table 2 [58].

Among these studies, the genus *Lippia* has received special attention over the last decade, being described in the next section.

## 4. Functional Ingredients based on Phenolic Compounds with Anti-Inflammatory Properties: Genus *Lippia*, A Case Study

### 4.1. Phytochemicals Present in Lippia Plants: Phenolic Compounds and Anti-Inflammatory Properties

The genus *Lippia* belongs to the Verbenaceae family, where about 250 herbaceous species of shrubs that may grow to as much as 3 m high, can be found. The most significant number of species grow in tropical areas of Central and South America and pantropical areas of Africa [65,66]. Nevertheless, they may also be found in Mediterranean areas [67]. These plants are popular for their characteristic fragrance. For this reason, species such as *Lippia origanoides* [68], *Lippia dulcis* [69] or *Lippia alba* [70] for example are commonly used as seasoning in food products.

In addition, some species have been used in traditional medicine to alleviate several ailments, such as stomach ache, fever, indigestion or joint pains [71,72,73]. The beneficial properties have been linked to phytochemicals present in these plants. Their composition and concentrations depend largely on geographical origin, soil and climate conditions, age, and portion of the plant [74,75]. Overall, phytochemicals present in *Lippia* plants could be classified in different categories based on their chemical structures as shown in Table 3 below.

Different non-phenolic compounds have been described in genus *Lippia*. Firstly, terpenes and terpenoids are characterized by their citric flavor and, consequently, they have been broadly used in several industries [92]. This heterogeneous group belongs to the essential oil fraction and they have been regarded as an interesting chemical group due to their antimicrobial, insecticide [93], anti-fungal [94], and anti-malarial properties [95]. A wide range of terpenes and terpenoids have been described in *Lippia* and hence divided into monoterpenes and monoterpenoids, sesquiterpens and sesquiterpenoids, all of which represent volatile compounds. The concentration of these compounds may vary depending on species [81]. Iridoids are a subgroup of monoterpenoids which are non-volatile compounds. In general, they present sugar moieties connected to an iridan core giving rise to the derivatives found in *Lippia*. Among the iridoids, loganic acid has been found in *L. citriodora* extracts and revealed to modulate several metabolic pathways [90]. In addition, several non-phenolic compounds have also been found which are related to oxipilins chemical groups which are plant hormones synthetized in stress conditions. In this minority group, derivative compounds of tuberonic acid [89] can be found.

**Figure 2:**
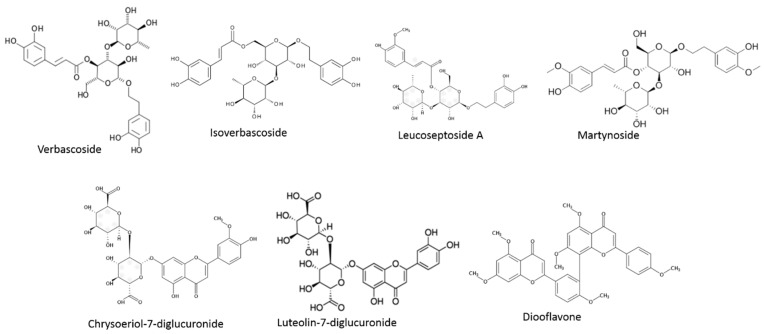
Representative phenolics in *Lippia* species.

Regarding phenolic compounds, two major groups are found in *Lippia* species (Figure 2). The most representative group is phenylpropanoids. This heterogeneous group is characterized by a phenol bone joined to a three-carbon propene tail. These molecules are primary units of a great number of polymers such as lignins and they provide ultraviolet light protection or may mediate pollination [96]. The most representative compound is verbascoside, also known as acteoside. But it can also be found in different isomers such as isoverbascoside or forsythoside A. All of them are caffeoyl phenylethanoid glycosides, where caffeic acid and hydroxytyrosol moieties are joined to a rhamnose. In addition, different verbascoside derivatives were found [89]. These compounds have been shown to provide hypotensive effects, neuroprotective, antioxidant, and anti-inflammatory effects [71]. This phenolic group has been broadly identified in *Lippia* plants, specifically in *L. dulcis, L. multiflora or L. citriodora* [97,98,99]. Nevertheless, other phenolics belonging to this group have been previously found in *Lippia* species. For instance, martynoside, leucoseptoside A, cistanoside F, verbasoside or campneoside I were also described in *L. citriodora* [89,90]. Furthermore, calceolarioside E was described in *L. alba* to provide anti-oxidant capacity [100].

On the other hand, the second major group widely studied in *Lippia* plants is flavonoids. The phenolic compounds merged into this group may be classified according to their structures. In this sense, flavones are the most representative in these botanicals. They may be found as 6-hydroxylated, methoxylated, and glycosylated flavonoids including their aglycone forms. For instance, luteolin may be found as 6-hydroxyluteolin, luteolin-7-diglucuronide or luteolin in *Lippia nodiflora, Lippia canescens* or *L. citriodora* [90,101]. One of the most abundant flavones is chrysoeriol which, as well as luteolin, may be found bound to a diglucuronide moiety, known as the most representative flavonoid in *L. citriodora* [77]. Additionally, other diglucuronic flavones such as apigenin or acacetin are also described [89]. Moreover, some flavanones have been characterized in *Lippia graveolens* such as naringenin, pinocembrin, and quercetin among others [88,102]. It has been claimed that some of these compounds possess hepatoprotective effects [103].

The phenolic composition of these plants may be changed according to climatic conditions likewise season and soil characteristics. Therefore, in a study performed on *L. alba*, the flavonoid concentration was higher during summer, whereas phenylpropanoid concentration was higher during winter [75]. Because of this phenolic composition *Lippia* have been claimed as an alternative to pharmacological drugs to treat different illnesses.

With regard to biological properties, the main effect of phenolic compounds has been linked to anti-oxidant and anti-inflammatory effects. In this way, the healthy potential of phenolics from *Lippia* and the emergence of new laws in food development have caused an enhancement of commercialization of diverse functional products based on this plant. For instance, PLX^®^ is a nutraceutical based on *L. citriodora* enriched in different concentrations of phenylpropanoids. Several assays performed on this food supplement have revealed that verbascoside was the most abundant compound followed by its isomer, isoverbascoside. Moreover, the most representative flavonoid was chrysoeriol-7-diglucuronide [89,90].

Some compounds of this extract have demonstrated the capacity to modulate several metabolic pathways related to energetic metabolism such as AMP-activated protein kinase (AMPK), mammalian target of rapamycin (mTOR) or inflammatory pathways like nuclear factor kappa-light-chain-enhancer of activated B cells (NF-κB). Indeed, it has been proved that a synergetic effect exists between phytochemicals contained in *Lippia* that modulate the expression of NF-κB in hypertrophic adipocytes. The level of this transcription factor decreased in the presence of lemon verbena extract. Moreover, an increase of adiponectin expression was also achieved. Hence, the anti-inflammatory action of adiponectin together with the decrease of NF-κB and a significant activation of AMPK may cause a regulation of different markers associated with carbohydrates and lipids metabolism, inflammation, oxidative stress, and insulin resistance [104].

Further studies demonstrated the individual effects of phytochemical from PLX^®^, revealing a significant effect of some iridoids (loganic acid and gardoside) and phenylpropanoids (mostly verbascoside), which could have a significant impact on AMPK modulation in in vitro assays [90]. In addition, it has also been proven that these compounds inhibit the production of cytokines such as TNF-α, IL-1β, and IL-6 [105]. These outcomes revealed that *L. citriodora* may help to prevent or manage several pathologies typified by disturbances on oxidative and inflammation pathways such as obesity or cancer.

Moreover, the effects of PLX^®^ have also been tested in in vivo models. Specifically, on rabbits it has achieved an enhancement of lipid markers decreasing total cholesterol by increasing high density lipoprotein cholesterol (HDLc) and decreasing low density lipoprotein cholesterol (LDLc), among other effects. Additionally, the anti-inflammatory properties of *L. citriodora* caused an improvement in liver and renal oxidative markers [106]. In a clinical trial, multiple sclerosis patients, where inflammation is one of the main contributing factors that induces this medical condition, were supplemented with PLX^®^ capsules in order to evaluate the anti-inflammatory ability of lemon verbena. Results reflected that *L. citriodora* extract might affect, depending on the patient clinical subtype, the cytokines profile of patients preventing or managing the physiological disorders induced by multiple sclerosis [73].

Likewise, an exhaustive exercise may induce oxidative stress and inflammation. This situation results in an increment of reactive oxygen species causing a disruption of myofibrillar structure and myofiber necrosis that cause higher muscle fatigue and distress. At this point, the intensity of exercise decreases whereas muscle recovery time increases, causing a reduction in performance. In this scenario, Buchwald-Werner et al., tested a new lemon verbena extract called Recoverben^®^ on active people. The outcomes revealed that 400 mg/day of *L. citriodora* extract resulted in significantly less muscle strength loss and an enhancement of muscle recovery after exhaustive exercise [58].

### 4.2. New Trends in Dietary Phenolics: Functional Ingredients and Nutraceuticals from the Lippia genus

The aging trend of the global population and the increasing incidence of chronic diseases have resulted in consumer demand for natural foods that may help to delay the onset of these ailments or improve their symptoms. In recent decades, the consumption of functional foods has soared, so much so that some ingredients with which they are made, such as phenolics, experienced a growth rate of 6.1% from 2012 to 2018 [107]. This trend has resulted in governments from all over the world passing new laws in order to regulate the natural sources whose bioactive compounds could be used as functional ingredients. Due to the beneficial properties provided by plants from the *Lippia* genus and the emerging interest in health-promoting products from consumers, there has been an increase in the demand for functional products and nutraceuticals. As previously mentioned, the European Union has listed extracts from plants of the *Lippia* genus, specifically *L. citriodora* extracts as a new food (CIR (EU) 2017/2470 of 20 December 2017), opening up a huge range of possibilities to food industries. These facts have promoted the optimization of different production processes with the purpose of retrieving bioactive compounds from natural sources.

The traditional extraction methods that have been applied to the recovery of bioactive compounds from botanical sources were based on solid–liquid extractions, including maceration, decoctions, and infusions. These kinds of technological procedures have been applied to industrial production of nutraceutical and functional ingredients from the *Lippia* genus. The food industry has addressed research and production to increase the level of phenolics in *Lippia* extracts, as well as the bioavailability in order to increase the biological and health effects.

Regarding retrieval of phenolics from *Lippia*, conventional extraction systems have been applied. Reviewing the literature available, Bilia et al. performed comparative macerations of *L. citriodora* and *Lippia officinalis* leaves. To achieve this goal, four different extractions were developed: two aqueous decoctions, an aqueous infusion, and an ethanolic maceration. The quantitative analysis showed that ethanolic maceration had reached a verbascoside yield higher than aqueous infusions and decoctions. In addition, from the two species analyzed, *L. citriodora* showed a higher concentration of verbascoside, and was claimed to be a great source of this bioactive compound [108]. Choupani et al., developed four different conventional extractions from *L. citriodora* leaves based on macerations using methanol, ethanol, water, and acetone where the extraction yields had values ranging from 3.4% to 10%. This study showed methanol to be the most efficient solvent to retrieve phenolic compounds [109]. Moreover, similar work has been performed using *L. alba* as the botanical source. Results displayed that temperature applied in aqueous extraction could generate the degradation of phenolic compounds. In addition, ethanolic extracts revealed higher concentrations of phenolic compounds [110].

Advanced extraction systems have increased their applications in the last decade. These procedures have instigated a technological and economic advance in the food industries, since they may provide enriched phenolic extracts that, after a drying process, could be used as a powder or added to food as functional ingredients. In addition, these non-conventional technologies reduce the environmental impact since they decrease the consumption of solvents, extraction time as well as energy consumption [111]. Indeed, these non-conventional technologies, also known as green technologies, have been developed to achieve several common objectives: (1) to extract specific bioactive compounds from botanicals, (2) to increase the concentration of targeted compounds in functional ingredients, (3) to improve the selectivity of extraction methods for target bioactive compounds, and (4) to supply a reproducible method that is independent of botanical source variations [112]. Some of these promising techniques are pressurized liquid extraction (PLE), supercritical liquid extraction (SFE) or microwave assisted extraction (MAE), among others. All of them are based on different ways of heat transmission or penetration inside the sample.

#### 4.2.1. Advanced Extraction Technique for Retrieval of Bioactive Compounds from the *Lippia* Genus

Thus, PLE is based on the combination of both high temperatures and pressures to achieve a huge variation in dielectric constant of solvents. These facts succeed in recovering a huge variety of phytochemicals [111]. This technique has acquired popularity due to the fact that it has proved to be more efficient in terms of time, amount of solvent and recovery of compounds than traditional extraction techniques. In addition, great extraction yields have been obtained when PLE has been applied. In fact, in a comparative study conducted on *L. citriodora*, PLE was shown to obtain greater extraction yield than hydro alcoholic macerations as well as phytochemical retrieval. Moreover, it has been proved to have higher selectivity than conventional extractions, maximizing the recovery of different chemical groups from *L. citriodora* leaves by a response surface methodology (RSM) [77].

Another advanced extraction technology that has been revealed to provide higher selectivity is SFE. This extraction system was applied to recover essential oil as well as phenolics from the *Lippia* genus. In comparative studies carried out on *L. alba*, SFE and several conventional extraction methods were compared and used to recover essential oils from plant leaves such as hydrodistillation or simultaneous distillation solvent extraction. These studies revealed that SFE provided a selective extraction of essential oil fraction from *L. alba* and it succeeded in recovering a wider range of volatile compounds than traditional extractions [113]. Moreover, a study conducted by Garmus et al., revealed that a sequential extraction of SFE, ethanol, and water provided phenolic enriched extracts from *Lippia sidoides* with higher antioxidant capacity [114]. These results suggested that SFE could be an interesting technique for flavor industries since it provides important amounts of essential oils. Although, SFE has also been shown to be an attractive method to obtain extract with large amounts of specific phenolic compounds as flavonoids [114].

Moreover, MAE has been revealed to be an advanced technology which may retrieve thermo labile compounds from the botanical source. MAE has been coupled with hydrodistillation techniques in order to retrieve the essential oil fraction from plant leaves. The outcomes revealed that this new technology provided higher reproducibility while needing less extraction times [74]. Conversely, MAE has been widely applied to retrieve the phenolic fraction from botanical sources [115,116] providing excellent results in terms of yield and phenolic profile. Moreover, MAE has been used to recover polar compounds from *L. citriodora* by using eutectic solvents, revealing itself to be an interesting alternative to achieve a selective extraction. These solvents comprise a mix of anionic and cationic species, such as choline chloride, lactic acid, ethylene glycol or urea. In this study, it might be seen how these kinds of solvents combined with MAE provided a selective extraction of some phenolics. For instance, the eutectic solvents formulated with choline chloride and lactic acid enabled a selective extraction of phenylpropanoids and flavonoids, with verbascoside and chrysoeriol-7-diglucuronide being the most recovered phenolics and the most promising green tailor-made solvents for retrieval phenolic from *L. citriodora* [117].

To establish which extraction system is more suitable, it should be continuously and critically scrutinized with important criteria, such as high retention capability, high flow rate, problem-free handling, and being environmentally-friendly, as well as physiological safety. Although several articles have pointed out the potential application of these advance extraction techniques, the main pilot-plant and industrial processes include resin chromatography, membrane technologies (filtration and reverse osmosis) and solid–liquid or liquid–liquid solvent extractions. Choosing the best system for recovering phenolic compounds from vegetable source may only be done after careful consideration of all the relevant parameter processes including scale-up. Over the last few years, SFE platforms have been introduced on an industrial scale as a selective extraction technique to obtain isolated compounds from vegetable sources. However, PLE and MAE remain on laboratory or pilot-plant level.

#### 4.2.2. Encapsulation Techniques

These obtained extracts which are enriched in phenolic compounds could be used to develop functional food such as ingredients. Nevertheless, some organoleptic features of phenolic compounds are not accepted by consumers due to their unpleasant taste. In addition, they have presented low bioavailability in the gastrointestinal tract. In this sense, it has been estimated that around 5–10% of the total dietary phenolic are absorbed in the small intestine and from them, aglycones present higher absorption rates [118]. Additionally, phenolic compounds bioavailability is affected by multiple factors such as low water solubility, the food matrix, its technological processes or the interactions produced through the gastrointestinal tract [119]. With the purpose of improving the bioavailability, bioaccessibility, and undesirable organoleptic aspects of phenolic compounds, several micro and nano-encapsulation methods have been developed in order to protect the bioactive compounds from these exogenous factors and to increase their bioavailability by a controlled release in the portion of gastrointestinal tract where their absorption is maximized as well as to mask any unpleasant features [120].

The encapsulation consists of creating vesicles or capsules that may range from nanometers to several millimeters in diameter whose morphology depends on the technological process, the core and wall materials. The process of manufacturing particles comprises the entrapment of the material to be encapsulated (core) by wall materials (encapsulating agents) ensuring that undesired materials are kept out and warranting that the particles are completely closed and avoid leakages [121]. In this scenario, the most widely used methods to encapsulate phenolic compounds are entrapment with liposomes, spray drying or freeze drying.

Firstly, the encapsulation with liposomes promote the entrapment of compounds of hydrophilic and hydrophobic nature at the same time. Although the number of bioactive compounds that may be loaded in these systems is very low, the bioavailability is profoundly high due to its amphipathic nature that facilitates the passage of bioactive compounds through the gut barrier. In this sense, liposomes are colloidal vesicles formed by a single or multiple lipid bilayer, generally phospholipids, attaining spherical nanoparticles between 30 nm to several micrometers improving their passage through intercellular spaces [122]. The characteristic nature of this membranous system allows the entrapment of several compounds such as protein or phenolic compounds into its aqueous, lipidic, and amphiphilic spaces [123,124] being usually used in the entrapment of different drugs. Additionally, Mazzacuva et al. reported that verbascoside obtained from *L. alba* was encapsulated in complex liposomes combined with cyclodextrin to improve the permeation of verbascoside through skin. The results showed that this complex system enabled the verbascoside release in skin [125].

Encapsulation by spray drying is a flexible, continuous, and economic procedure to attain high quality particles. This technique is not only used in the entrapment of phenolics, but also to encapsulate several additives and flavors [126]. The result of this process is a homogenous powder with a size of particle ranging between 10–100 µm [127]. Several polysaccharides have been used as wall materials to encapsulate polar compounds such as maltodextrins, inulin or alginate [128,129,130]. Indeed, β-cyclodextrin has been used to encapsulate essential oils from *L. graveolens* by spray drying, achieving an encapsulation efficiency of up to 81%. Moreover, these particles succeeded in preserving de-antimicrobial activity and to improve the antioxidant activity of these essential oils since the wall material protected essential oils [131].

On the other hand, the freeze-drying principle is based on the thermodynamic properties of water, getting a result by modifying the surrounding pressure and the sublimation of the water inside the material near to room temperature. This is a versatile technique which achieves a dehydration of the material avoiding thermal degradation of the sensitive compounds. Freeze drying is also known as lyophilization, and the particles obtained present uncertain forms [44]. The biggest drawbacks to this methodology are the long process times and the low encapsulation efficiency [132]. Although, reports did not find anything about encapsulation by lyophilization of either phenolics or essential oils from *Lippia*, this technique has been revealed as a better encapsulation process to entrap essential oils from plant rather than spray drying [133]. Another study revealed how freeze-drying encapsulation succeeded in providing more efficient powder with higher antioxidant capacity than spray drying [134].

Although spray drying has been used to encapsulate essential oils from *Lippia*, neither freeze drying nor spray drying have been performed to encapsulate polyphenols from these plants. Nevertheless, these encapsulation techniques may be an alternative to improve the bioavailability efficiency of phenolic extracts obtained from *L. citriodora* and even to apply to current commercial extracts to implement the bioactivity of phenolics contained in them.

In summary, these extraction and encapsulation systems have opened up a huge variety of opportunities in functional food development, optimizing the resource utilization and energy consumption, as well as improving the bioavailability of bioactive compounds obtained from natural sources. These facts have resulted in botanicals potentially being considered as an interesting alternative to treat different diseases.

## 5. Conclusions

Undoubtedly, the beneficial effects of phenolic compounds from natural sources have been broadly tested. In this sense, several studies have revealed that phenolic compounds have a great potential modulating ability on pro-inflammatory mediators, giving these phytochemicals a significant role in the prevention or management of many chronic diseases. Although more studies on the processes derived from these chronic diseases, such as inflammatory disorders, are necessary, *L. citriodora* has been claimed as a natural source of phenolic compounds with anti-inflammatory properties. This botanical has been used to develop several food supplements (PLX^®^ and Recoverben^®^) and has been tested on humans in order to prove the anti-inflammatory or anti-obesogenic effects with interesting results. On the other hand, the emergence of new extraction technologies may enable the retrieval of bioactive compounds from botanical sources opening up a new research field in the utilization of plant-derived phenolics. Additionally, to improve the bioavailability and technological processes of phenolic compounds, several microencapsulation strategies have been evaluated. These methodologies provide some benefits in functional ingredients during functional food formulation. All of these outcomes could confirm the potential use of *L. citriodora* as a functional ingredient and its utilization as an alternative to manage pro-inflammatory states. Nevertheless, this report reflected that more studies on *L. citriodora* are needed in order to evaluate the extraction kinetics of phenolic compounds in MAE and SFE, as well as to ascertain the behavior of these compounds during microencapsulation processes.

## Figures and Tables

**Table 1:** Modulation of inflammatory response of phenolic compounds tested on in vivo models.

Class	Phenolic Compounds	Biomarkers Studied	Mechanisms	Doses	Model	Reference
Phenolic acids	*p*-coumaric acid	IL-12, TNF-α, and IL-1β	Decreased TNF-α expression Decreased circulating immune complexes	100 mg/kg body weight	Adjuvant-induced arthritic rats	[29]
	Chlorogenic acid	NO	Decreased the activity of iNOS	5, 20, and 50 mg/kg body weight	LPS-induced acute lung injury rats	[30]
	Rosmarinic acid	TNF-α, Il-1β, and IL-6	Decreased neutrophil activity Decreased MMP-9 activity Modulation of NF-κB	10, 25, and 50 mg/kg body weight	Carrageenin-induced paw oedema rat	[31]
	Ferulic acid	iNOS, COX-2, TNF-α, and IL-1β	Decreased JNK/NF-κB	20 mg/kg body weight	LPS-induced neuroinflammation rats	[32]
	Verbascoside	TNF-α, IL-1β, and IL-6	Decreased IκBα Decreased NF-κB Decreased IKK-α Decreased IKKβ	30 and 60 mg/kg body weight	LPS-induced acute lung injury rats	[33]
Flavonol	Rutin	NOS-2, NF-κB, IkBa, and IL-17	Increased NF-κB Increased IκBα phosphorylation	10, 20, and 40 mg/kg body weight	CFZ-induced nephrotoxicity rats	[34]
Flavonol	Quercetin	IL-1β, IL-6, and TNF-α	Decreased LPO, NF-κβ, and TNF-α.	10, 20, 25, 40, and 50 mg/kg body weight	Streptozotocin-nicotinamide induced diabetic rats Induced inflammation by radiation mice	[35,36]
Flavonol	Kaempferol	IL-1β, IL-6, TNF-α, MCP-1, and ICAM-1	Decreased HMGB1/TLR4 inflammatory pathway	20 and 50 mg/kg body weight	LPS-induced striatum injury mice	[37]
Flavanol	Kaempferol-3-O-glucorhamnoside	TNF-α, IL-6, IL-1β, and PGE2	Decreased NF-κB and MAP kinase phosphorylation	50, 100 or 200 μg/kg	*Klebsiella pneumoniae* infected mice	[38]
Flavone	Luteolin	IL-1β, NO, PGE2, TNF-α, NOS, COX-2,MMP-1, MMP-2, MMP-3, MMP-8, and MMP-9	Decreased NF-κB	10 mg/kg body weight	MIA-induced osteoarthritis mice	[39]
Flavone	Luteolin-7-glucoside	IL-1β, TNF-α, iNOS, and COX-2	Decreased NF-κB Increased PPARγ Increased Nrf2	20, 40, 80 mg/kg body weight	Cerebral ischemia-reperfusion induced neuroinflammation rats	[40]
Flavone	Acacetin	TNF-α, IL-1β	Decreased TNF-α Increased HO-1	50 mg/kg body weight	LPS-induced acute lung injury rats	[41]
Flavan-3-ol	Epicatechin	TNF-α, iNOS, and IL-6	Decreased TLR4 upregulation Decreased NOX activation Decreased NF-kB activation.	80 mg/kg body weight	LPS-induced renal inflammation rats	[42]
Flavanol	Epigallocatechin gallate	TNF-α, IL-1β, IL-6, CINC-3 and iNOS	Increased Nrf2 Increased HO-1	40 mg/kg body weight	Fluoride induced lung oxidative stress rats	[43]
Ellagitannin	Ellagic acid	NO, caspase-3, MMP-9, IL-1β	Decreased NF-κB	50 mg/kg body weight	FCA-induced arthritis rats	[44]

CFZ: Carfilzomib-induced oxidative stress; CINC-3: Cytokine-induced neutrophil chemoattractant; COX-2: Cyclooxygenase-2; FCA: Freund’s complete adjuvant; HMGB1: High mobility group box 1; HO-1: Heme oxygenase 1; ICAM-1: Intercellular cell adhesion molecule-1; IκBα: Inhibitory kappa-B alpha; IKK-α: Nuclear factor kappa-B kinase-α; IKKβ: Nuclear factor kappa-B kinase-β; IL: Interleukin; iNOS: Inducible nitric oxide synthase; JNK: c-Jun N-terminal kinases; LPS: Lipopolysaccharide; LPO: Lipid peroxidation; MAP: Mitogen-activated protein kinase; MCP-1: Monocyte chematotactic protein-1; MIA: Monosodium iodoacetate induced osteoarthritis; MMP-9: Metalloproteinase-9; Nrf2: Increased NF-E2-related factor; NO: Nitric oxide; PGE2: Prostaglandin E2; PPARγ: Peroxisome proliferator-activated receptor; TLR4: Toll-like receptor 4.

**Table 2:** List of available nutraceuticals to manage inflammatory events.

Source	Composition	Effect	Reference
***L. citriodora***	Mainly verbascoside and other phenylpropanoids	Anti-inflammatory and reducing muscle damage after sport	[58]
**Not revealed**	Phytosterols, RYR, hydroxytyrosol, and vitamin E	Anti-inflammatory in patients with hypercholesterolemia	[59]
***Acacia catechu* and *Morus alba***	Catechin, stilbenes, and flavonoids	Anti-arthritis effects	[60]
**Olive oil**	Oleocanthal, ligstroside aglycone, docosahexaenoic, and eicosapentaenoic acids	Anti-osteoarthritis	[61]
**Chamomile**	Apigenin, apigenin-7-glucoside, and apigenin-7-(6-acetil)glucoside.	Inflammatory in bowel diseases	[62]
**Pomegranate fruit**	Mainly ellagic acid	Anti-inflammatory in obese patients	[63]
***Brassica oleracea***	Anthocyanins	Inflammatory in bowel diseases	[64]

RYR: red yeast rice.

**Table 3:** Representative phytochemicals found in aerial parts of *Lippia* genus plants.

Chemical Group	Compound	Species	Reference
**Iridoids**	Carioptoside	*Lippia alba*	[76]
	Gardoside	*L. citriodora*	[77]
	Durandoside I	*L. citriodora*	[77]
	Hydroxyl campsiside	*L. citriodora*	[77]
	Ixoside	*L. citriodora*	[77]
	Lipedoside A I	*L. citriodora*	[77]
	Lippioside I	*L. citriodora*	[77]
	Lippioside II	*L. citriodora*	[77]
	Loganic acid	*L. alba*	[76]
	Manuleoside H	*L. citriodora*	[77]
	Myxospyroside	*L. citriodora*	[77]
	Shanzhiside	*L. alba*	[76]
	Secologanin	*L. alba*	[76]
	Secoxyloganin	*L. alba*	[76]
	teucardoside	*L. citriodora*	[77]
	Theviridoside	*Lippia javanica*	[76]
	Theveside	*L. citriodora*	[77]
**Terpenes**	Camfor	*L. citriodora*/*javanica*	[78,79]
	Caryophyllene	*L. citriodora*/*javanica*	[67,79]
	Carvacrol	*L. citriodora*/*origanoides*	[67]
	Cimonene	*L. citriodora*	[78]
	Citral	*L. citriodora*/*dulcis*	[80,81]
	Citronelal	*L. citriodora*	[5]
	Curcumene	*L. citriodora*	[82]
	Eucalyptol	*L. javanica*	[79]
	Escualen	*L. citriodora*	[5]
	Geranial	*L. citriodora*/*javanica*	[67,79]
	Geraniol	*L. citriodora*/*javanica*	[67]
	Heptacosanol	*L. citriodora*	[82]
	Ipsdienone	*L. javanica*	[79]
	Limonene	*L. citriodora*/*javanica*	[79,83]
	Linalool	*L. citriodora*/*javanica*	[79,84]
	Myrcenone	*L. citriodora*/*javanica*	[79,84]
	Neral	*L. citriodora*	[5]
	Nerol	*L. citriodora*	[85]
	Nonenal	*L. citriodora*/*javanica*	[67,79]
	Sabinene	*L. citriodora*/*javanica*	[78,79]
	Spathulenol	*L. javanica*	[79]
	Thymol	*L. citriodora*/*origanoides*	[83]
	α-Terpineol	*L. citriodora*	[5]
	β-Caryophyllene	*L. citriodora*/*dulcis*	[81,83]
	β-Cymene	*L. citriodora*	[83]
	*p*-Cymene	*L. origanoides*	[81]
	γ-Terpinene	*L. citriodora*/*javanica*	[67,79]
**Flavonoids**	Acacetin-7-diglucuronide	*L. citriodora*	[86]
	Apigenin	*L. citriodora*/*graveolens*/*javanica*	[79]
	Apigenin-7-diglucuronide	*L. alba*/*citriodora*	[86,87]
	Carssifolioside	*L. javanica*	[79]
	Chrysoeriol	*L. citriodora*/*javanica*	[67,79]
	Chrysoeriol-7-diglucuronide	*L. citriodora*	[86]
	Cirsiliol	*L. citriodora*	[67]
	Cirsimaritin	*L. citriodora*	[67]
	Dimethylscutellarein	*Lippia graveolens*	[88]
	Diosmetin	*L. citriodora*	[67]
	Eriodictyol	*L. graveolens*	[88]
	Eriodictyol -7-glucoside	*L. graveolens*	[88]
	Eupafolin	*L. citriodora*	[67]
	Eupaforin	*L. citriodora*	[67]
	Eupatorin	*L. javanica*	[79]
	Galangin	*L. graveolens*	[88]
	Genkawin	*L. javanica*	[79]
	Hipidulin	*L. citriodora*	[67]
	Hydroxyluteolin	*L. citriodora*/*graveolens*	[88]
	Hydroxyluteolin 7-O-hexoside	*L. graveolens*	[88]
	Hydroxyluteolin 7-O-rhamnoside	*L. graveolens*	[88]
	Isothymusin	*L. javanica*	[79]
	Jaceosidin	*L. citriodora*	[86]
	Luteolin	*L. citriodora*/*javanica*	[67,79]
	Luteolin-7-glucoside	*L. alba*/*citriodora*/*graveolens*	[86,87,88]
	Methylscutellarein	*L. graveolens*	[88]
	Methoxylutrolin	*L. javanica*	[79]
	Naringenin	*L. graveolens*	[88]
	Nepetin	*L. citriodora*	[67]
	Nepitrin	*L. citriodora*	[67]
	Quercetin	*L. citriodora*/*graveolens*	[67,88]
	Pectolinarigenin	*L. citriodora*	[67]
	Phloretin	*L. graveolens*	[88]
	Phloretin-6-glucoside	*L. graveolens*	[88]
	Pinocembrin	*L. graveolens*	[88]
	Sakuranetin	*L. graveolens*	[88]
	Salvigenin	*L. citriodora*/*javanica*	[67,79]
	Scutellarein	*L. graveolens*	[88]
	Scutellarein-7-hexoside	*L. graveolens*	[88]
	Taxifolin	*L. graveolens*	[88]
	Tricin	*L. javanica*	[79]
**Phenylpropanoids**	Calceolarioside E	*L. alba*	[87]
	Cistanoside F	*L. alba*/*citriodora*	[86,87]
	Decaffeoylverbascoside	*L. alba*	[87]
	Eukovoside	*L. citriodora*	[89]
	Hydroxy-verbascoside	*L. citriodora*	[89]
	Hydroxy-isoverbascoside	*L. citriodora*	[89]
	Forsythoside A	*L. citriodora*	[89]
	Forsythoside B	*L. alba*	[87]
	Isonuomioside	*L. alba*	[87]
	Isoverbascoside	*L. citriodora*/*graveolens*/*javanica*	[79]
	Lariciresino glucopyranoside	*L. citriodora*	[77]
	Leucoseptoside	*L. citriodora*	[90]
	Lippianoside B	*Lippia tryiphylla*	[91]
	Martynoside	*L. citriodora*	[90]
	Osmanthisude B	*L. citriodora*	[77]
	Verbascoside	*L. citriodora*/*graveolens*/*javanica*	[79,88]
	Verbascoside A	*L. citriodora*	[77]
	Verbascoside	*L. citriodora*	[89]
**Oxipilins**	Tuberonic acid glucoside	*L. citriodora*	[89]
